# Semifield evaluation of autodissemination systems in *Aedes notoscriptus* and *Culex quinquefasciatus* and laboratory analysis of In2Care formulation effects

**DOI:** 10.1093/jme/tjag078

**Published:** 2026-06-02

**Authors:** Véronique Paris, Nicholas Bell, Ary A Hoffmann

**Affiliations:** Pest and Environmental Adaptation Research Group, School of BioSciences, Bio21 Institute, University of Melbourne, Parkville, VIC, Australia; Pest and Environmental Adaptation Research Group, School of BioSciences, Bio21 Institute, University of Melbourne, Parkville, VIC, Australia; Pest and Environmental Adaptation Research Group, School of BioSciences, Bio21 Institute, University of Melbourne, Parkville, VIC, Australia

**Keywords:** Mosquito control, Autodissemination, Pyriproxyfen

## Abstract

Urban mosquito control needs targeted tools that suppress population breeding in cryptic container habitats while reducing reliance on broad-spectrum insecticides. Autodissemination devices exploit oviposition behavior to transfer the insect growth regulator pyriproxyfen to larval sites and may be enhanced by adulticidal agents such as the entomopathogenic fungus *Beauveria bassiana*. We compared semifield efficacy of In2Care^®^ Mosquito Stations and INZECTO Mosquito OviTraps + INZECTO pyriproxyfen chips against *Aedes notoscriptus* and *Culex quinquefasciatus*. We subsequently quantified the adult survival effects of pyriproxyfen, *B. bassiana*, and their combined formulation (In2Mix) in *Ae. notoscriptus*. Semifield trials used 4 × 4 m mesh tents in replicate blocks; 50 females per tent were exposed to In2Care stations, INZECTO OviTraps + INZECTO pyriproxyfen chips, or untreated controls with four surrounding oviposition cups. Cup water was bioassayed for emergence inhibition and analyzed with beta-binomial GLMMs. Adult females were force-exposed to treated nettings (pyriproxyfen-only, *B. bassiana*-only, In2Mix, control) and survival was analyzed with Cox regression. In semifield trials, In2Care reduced adult emergence relative to controls (logit estimate = 1.892, *P* < 0.0001) and was more effective than INZECTO (estimate = −0.990, *P* = 0.024); INZECTO also reduced adult emergence compared to controls (estimate = 0.902, *P* = 0.041). In laboratory assays, *B. bassiana* substantially increased mortality hazard (HR = 7.26). The In2Mix treatment produced a comparable effect (HR = 8.64), suggesting that most of the observed mortality in the combined treatment is driven by the *B. bassiana* component. Pyriproxyfen alone also significantly elevated mortality risk relative to the control, although the effect size was more moderate (HR = 2.35).

## Introduction

Mosquito-borne diseases remain a major global health challenge, with transmission risk increasingly driven by urbanization, climate change, and the emergence of insecticide resistance. Urban expansion increases the availability of artificial container habitats and human–vector contact (Tatem et al. 2013, Trewin et al. 2020), while rising temperatures and altered rainfall patterns extend mosquito breeding seasons and geographic ranges (Campbell et al. 2015). At the same time, resistance to conventional insecticides has become increasingly prevalent across multiple mosquito taxa, undermining the long-term effectiveness of chemical control programs (Bhatt et al. 2013, Dusfour et al. 2019). Together, these pressures necessitate the development of diversified and community-informed mosquito management strategies capable of targeting cryptic, transient, or spatially dispersed larval habitats.

One such approach is autodissemination, which exploits adult mosquito egg-laying behavior to deliver larvicides to breeding sites that are otherwise difficult to locate or treat directly. In this strategy, adult mosquitoes acquire insect growth regulators (IGRs), most commonly pyriproxyfen, from oviposition devices and subsequently transfer them to larval habitats during oviposition or contact with water bodies. Even minute quantities of pyriproxyfen are sufficient to disrupt juvenile hormone regulation and inhibit adult emergence, making it particularly well suited to dissemination via adult mosquitoes (Itoh et al. 1994, Devine et al. 2009). A growing body of laboratory, semifield, and field studies has demonstrated that autodissemination can substantially suppress mosquito populations, particularly in container-breeding mosquito species (Unlu et al. 2020, Buckner et al. 2021, Kancharlapalli et al. 2021, Paris et al. 2023, Elfekih et al. 2025).

An example of this strategy is the In2Care^®^ Mosquito Station, which integrates pyriproxyfen with the entomopathogenic fungus *Beauveria bassiana* (http://www.In2Care.org). This dual-action formulation is designed to reduce adult mosquito longevity through *B. bassiana* while simultaneously inhibiting larval development via pyriproxyfen transfer. Gravid females are attracted to the station as an oviposition site, where eggs laid within the station fail to eclose as biting adults due to pyriproxyfen-induced alterations to their development (Unlu et al. 2020), functioning like a lethal oviposition trap. During egg-laying, adults become contaminated with pyriproxyfen and subsequently transfer it to other breeding sites. Field evaluations have reported significant reductions in adult abundance and larval emergence following In2Care deployment, supporting its utility as a population suppression tool (Buckner et al. 2017, 2021, Brisco et al. 2023, Paris et al. 2023).

Another tool is the INZECTO Mosquito OviTrap combined with INZECTO pyriproxyfen chips, which also functions primarily as a lethal oviposition trap, targeting container-breeding mosquitoes through oviposition attraction (http://www.inzecto.com). Larvae developing within the trap are exposed to a slow-release formulation of pyriproxyfen that prevents successful emergence. However, unlike In2Care, the INZECTO system is not explicitly designed to promote adult contamination and onward transfer of pyriproxyfen. As a result, it remains unclear whether mosquitoes interacting with INZECTO OviTraps containing pyriproxyfen chips can contribute meaningfully to pyriproxyfen dissemination beyond the trap itself or whether its efficacy is largely restricted to direct larval control within the device.

The effectiveness of autodissemination-based interventions is strongly influenced by mosquito oviposition behavior. *Aedes notoscriptus*, a container-breeding species and emerging disease vector in Australia, exhibits pronounced skip-oviposition, distributing eggs across multiple sites in small batches sites (Colton et al. 2003). This behavior is thought to enhance the spread of pyriproxyfen among larval habitats and has been associated with successful autodissemination in other *Aedes* species (e.g., Buckner et al. 2017, 2021, Unlu et al. 2020). In contrast, *Culex quinquefasciatus*, an important nuisance mosquito and potential arbovirus vector, typically lays eggs in rafts on water surfaces and displays less consistent oviposition site fidelity (Suleman and Shirin 1981). These behavioral differences may limit its capacity to acquire and disseminate larvicides, potentially reducing the effectiveness of autodissemination strategies for this species.

In this study, we compare the performance of In2Care stations and INZECTO OviTraps + pyriproxyfen chips in controlling *Ae. notoscriptus* and *Cx. quinquefasciatus* under semifield conditions. We assess pyriproxyfen-mediated inhibition of larval development using water samples collected following exposure of both species to the respective products. In addition, to disentangle the individual and combined effects of the active ingredients in the In2Care formulation, we separately evaluate the impacts of *B. bassiana* and pyriproxyfen on *Ae. notoscriptus* survival as well as their potential synergistic effect in the In2Mix.

## Methods

### Permits

This work was performed under APVMA Small-scale Trial Permit PER7250. This allows trials to be conducted to generate data relating to efficacy, residues, crop or animal safety, or other scientific information outside the confines of a research facility where the size of the trial annually does not exceed the following: a total of 5 ha nationally, with a maximum of 1 ha in any one jurisdiction in the case of any food and/or fiber field crop (https://permits.apvma.gov.au/PER7250.PDF). The active ingredients in In2Mix sachets were imported under biosecurity permit 0006570560 from the Australian government Department of Agriculture, Fisheries and Forestry.

### Mosquito Rearing

All laboratory experiments in this study used mosquitoes from laboratory colonies of *Ae. notoscriptus* and *Cx. quinquefasciatus*. Both *Ae. notoscriptus* and *Cx. quinquefasciatus* colonies were established from field collections in Brisbane, Australia. Colonies were maintained in a temperature-controlled insectary at 26 °C ± 1 °C and 50–70% relative humidity, with a 12-h light: dark photoperiod, following the protocol outlined by Ross et al. (2017). Adults were provided with constant access to a 10% sucrose solution, which was replaced with water one day before blood feeding. Females were blood-fed on a human volunteer’s arm once per generation to initiate egg-laying (University of Melbourne Human Ethics approval 0723847).

Egg collection differed between species: *Ae. notoscriptus* eggs were collected on sandpaper strips placed in cups containing larval water, while *Cx. quinquefasciatus* egg rafts were collected directly from the water surface. *Ae. notoscriptus* eggs (<1 week old) were induced to hatch by adding 5–10 grains of active dry yeast to reverse osmosis water, whereas *Cx. quinquefasciatus* eggs hatched in hay-infused water. Larvae of both species were reared at a density of ∼500 larvae per tray (40 cm × 30 cm × 6.5 cm) with 4 L of water and fed 0.05 mg per larva per day of Hikari tropical sinking wafers (Kyorin food, Himeji, Japan). Pupae were separated by sex based on size and morphology, and adult sex was confirmed within 24 h of emergence. Adult mosquito density was maintained at ∼500 per 27 L BugDorm-1 cage (MegaView Science Co., Ltd., Taichung, Taiwan).

### Comparative Semifield Autodissemination Experiments

#### Semifield Experimental Design

Semifield experiments were conducted to evaluate whether (i) INZECTO Mosquito OviTraps + INZECTO pyriproxyfen chips and (ii) In2Care Mosquito Stations inhibited adult emergence and whether mosquitoes could disseminate pyriproxyfen from devices to surrounding oviposition containers. *Aedes notoscriptus* and *Cx. quinquefasciatus* were tested in separate tents but in the same replicate blocks.

**Fig. 1.**
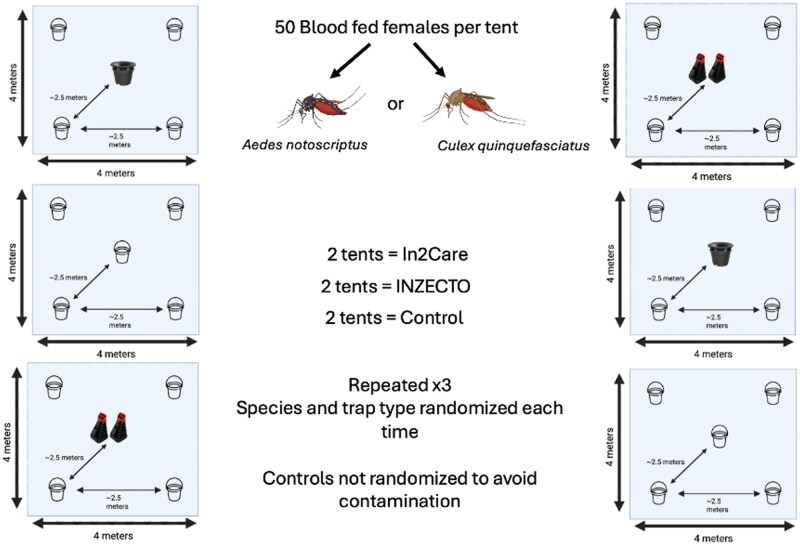
Experimental layout of semifield autodissemination trials: Each experimental block included six 4 × 4 m tents, with 50 blood-fed *Aedes notoscriptus* or *Culex quinquefasciatus* females released per tent. Two tents per block were assigned to INZECTO OviTraps, two were assigned to In2Care stations, and two served as untreated controls. Each tent contained four oviposition cups spaced ∼2.5 m apart around a central treatment station. Species were randomized within each block (×3 blocks), while control tents remained fixed to avoid cross-contamination with pyriproxyfen. Control tents received an additional ovitrap instead of a treatment station. Created resources with BioRender.com, inzecto.com and in2care.org.

Each experimental block comprised six mesh tents (4 × 4 m; Biomesh Insect Screening, Redpath Ideal, Australia) housed within a single polytunnel: two tents per block were assigned to INZECTO, two to In2Care, and two served as untreated controls (Fig. 1). In each tent, 50 blood-fed females of a single species were released. Female mosquitoes of each species were fed one day prior to release into the tents to ensure they were well fed, actively seeking oviposition sites, and provided with a recovery time after handling and transport. Each tent contained four oviposition containers positioned approximately 2.5 m apart around the central treatment device position. Oviposition containers consisted of 375 mL clear takeaway cups placed inside black buckets and filled with 300 mL tap water. In control tents, an additional oviposition container was placed in the central position to match the physical layout of treatment tents. In INZECTO tents, two traps were deployed per tent to account for the higher recommended deployment density (one trap per 200 m^2^), which is approximately double that of In2Care (one station per 400 m^2^); accordingly, In2Care tents contained a single station per tent. A cup containing a cotton ball soaked with a 10% sucrose solution was placed inside each tent to provide a sugar source for mosquitoes.

Microclimatic conditions within the polytunnel were recorded using an ELITECH data logger (EL-USB series; ELITECH Technology Inc.), positioned at the center of the polytunnel to capture in situ temperature variation throughout the experiment. External ambient temperature data over the corresponding period were obtained from the Australian Bureau of Meteorology (BOM), using the nearest available weather station to the University of Melbourne Burnley Campus (at Flemington Racecourse). Daily maximum and minimum temperatures from both datasets were aligned to the experimental period.

Photoperiod was calculated for each experimental date using solar position modeling with the R package suncalc (Thieurmel et al. 2019), based on the geographic coordinates of Melbourne (37.81°S, 144.96°E), with daylength derived from sunrise and sunset times.

Species and treatment assignment were randomized among non-control tents within each block; control tents remained in fixed positions across blocks to minimize the risk of pyriproxyfen cross-contamination. Mosquitoes were left undisturbed for five days to facilitate egg-laying.

On day 5, all takeaway containers from within the oviposition cups were collected and sealed individually in separate bags for transport. Water samples were also collected directly from the In2Care station reservoir and from each INZECTO trap to serve as positive controls for assay performance. Fresh devices and consumables (INZECTO traps and chips, In2Care stations/refills, buckets, and cups) were used for each block.

#### In2Care Stations and Setup

The In2Care mosquito station is constructed of polyethylene and comprises several components, including a lid, central tube, detachable interface, and a reservoir filled with 3.5–4.5 L of water infused with two yeast tablets to attract gravid mosquitoes. A floating platform moves up and down the central tube in response to the water level, providing a resting place for adult mosquitoes. This platform is equipped with a statically charged gauze strip treated with a powder formulation containing the In2Mix. When adult mosquitoes land on the gauze, the actives from the powder are transferred to them. The In2Mix powder is sealed in an aluminum refill sachet, each containing 0.5 g of formulation composed of approximately 74.03% pyriproxyfen and 10.00% *B. bassiana* strain GHA, with a minimum of 4.5 × 10^9^ viable spores per gram (https://www.in2care.org). Mosquitoes can be in contact with water at the station and lay eggs there, while immature development is stopped at the pupal stage through the effects of pyriproxyfen.

#### INZECTO OviTrap + INZECTO Pyriproxyfen Chips Setup

INZECTO OviTraps were prepared following the manufacturer’s instructions. Traps were filled with room-temperature tap water to the indicated maximum fill level, as marked by the water relief spout/fill indicator. A single INZECTO pyriproxyfen chip was added to each trap to initiate slow release of the formulation upon contact with water. Mosquitoes can be in contact with water at the station and lay eggs there, while immature development is stopped at the pupal stage through exposure to pyriproxyfen.

### Laboratory Testing of Pyriproxyfen Autodissemination

To assess the presence and residual activity of PPF in water collected from the semifield experimental setup, emergence inhibition was measured using *Ae. notoscriptus* larvae.

Before setting up containers, we passed each water sample through fine mesh to remove any eggs or debris. Fresh mesh was used for each sample to prevent cross-contamination. A 250 mL aliquot of the filtered water was poured into a clean plastic container and 25 laboratory-reared L2 *Ae. notoscriptus* larvae were introduced along with one pellet of TetraMin tropical fish food (Tetra, Melle, Germany). Containers were covered with fine mesh stockings to prevent adult escape.

Control samples were always prepared first to minimize the risk of cross-contamination. All containers were maintained in a 26 °C climate-controlled cabinet under a 16:8 light: dark cycle. Emergence was monitored daily until all larvae had either died or developed. Larvae were fed as required. Only fully formed, free-flying adults were counted as having successfully eclosed. The data for the autodissemination experiment can be found in Supplementary Table S1.

### Comparative Experiment of the Different Components Used in the In2Mix

To disentangle the individual effects of the actives in the In2Mix (i.e., *B. bassiana* and pyriproxyfen) on the survival of *Ae. notoscriptus*, as well as possible synergistic effects of both, we conducted additional laboratory experiments. All mosquitoes used were adults, seven days post-eclosion, and had been allowed to mate. We exposed 25 females at one time to one of the following nettings:

1. Pyriproxyfen-only (containing approximately 0.2 g of 74.03% pyriproxyfen)
2. *B. bassiana*-only (containing approximately 0.2 g with 10% *B. bassiana* spores)
3. In2Mix (containing approximately 0.2 g of In2Mix powder with 74.03% pyriproxyfen and 10% *B. bassiana* spores)

#### Untreated Netting (Used as a Control)

The exposures were conducted using the WHO standard forced exposure bioassay (World Health Organization 2006). We prepared eight replicates of 25 females for each treatment and control group. Each replicate group was provided with a 10% sucrose solution, which was refreshed as needed. Mosquitoes that died within 1 h of the experiment being setup were excluded from all analyses.

Survival was monitored daily until all individuals in each treatment cohort had died. Mosquitoes were considered dead if they showed no movement after being touched by forceps. The survival data can be found in Supplementary Table S2.

### Statistical Analysis

#### Semifield Species and Tool Comparison

All analyses were performed in R Studio v2024.04.1 (R Core Team 2021). Larval emergence was analyzed using generalized linear mixed-effects models in R (v4.4.0) with the glmmTMB package (Brooks et al. 2017). The response variable was the number of adults emerged per replicate, modeled as a binomial outcome, representing successful development out of a fixed number of larvae per cup (*n* = 25). Because preliminary diagnostics indicated extra-binomial variation, a beta-binomial error distribution with a logit link was used for final inference.

Fixed effects included treatment and species and their interaction, which was initially evaluated. Experimental block was included as a random intercept to account for spatial or temporal clustering within the experimental design. Model assumptions were assessed using simulation-based residual diagnostics implemented in the DHARMa package (Hartig 2024). No evidence of overdispersion or model misfit was detected (dispersion parameter close to 1; non-significant dispersion test), indicating that the beta-binomial model adequately accounted for dispersion in the data and was therefore used for all analyses.

Model selection was conducted using Akaike’s Information Criterion (AIC). A candidate set of nested models was compared, including: (1) treatment × species interaction, (2) additive treatment + species effects, (3) treatment only, (4) species only, and (5) an intercept-only model. The additive model (treatment + species) had the lowest AIC and was selected as the best fitting model.

#### Comparative Experiment of the Different Components Used in the In2Mix

To investigate whether mosquitoes infected with *B. bassiana*, pyriproxyfen and In2Mix had a shorter lifespan than control groups, we performed a Cox regression survival analysis using the “survival” package in R (Therneau and Lumley 2015). The treatment provided the explanatory variable, while the replicate was added as a random factor.

## Results

### Semifield Autodissemination Comparisons

**Fig. 2.**
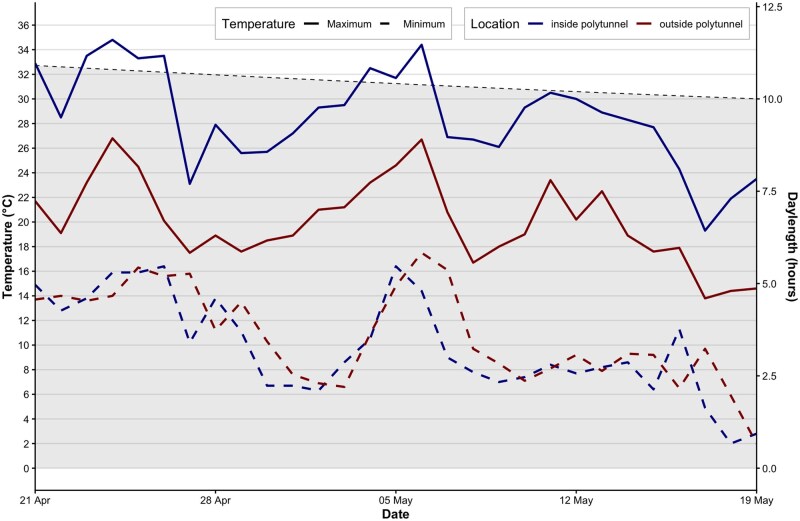
Daily minimum and maximum temperatures recorded inside the polytunnel during the experiment and under ambient conditions at a nearby weather station, alongside corresponding daylength. Solid lines represent maximum temperatures, and dashed lines represent minimum temperatures, with blue indicating conditions inside the polytunnel and red indicating external ambient conditions. The grey dashed line shows the seasonal decline in daylength over the study period.

Environmental conditions inside the polytunnel during the experimental period were warmer than ambient conditions, although minimum temperatures were similar (Fig. 2). Minimum temperatures were broadly consistent with typical late summer–autumn ambient conditions in Melbourne (BOM), with mean daily minimum temperatures of around 10 °C, although maximum temperatures averaging 28.5 °C inside the polytunnel were several degrees higher than the long-term average of around 20 °C. The mean daylength across the experimental period was 10.4 h (Fig. 2).

**Fig. 3.**
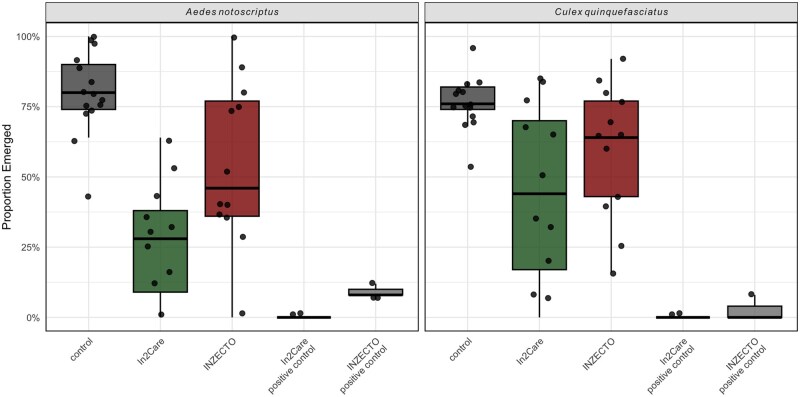
Box plots of the proportion of emerged *Aedes notoscriptus* from water samples collected in semifield autodissemination trials plotted by species (left: *Culex quinquefasciatus*; right: *Aedes notoscriptus*). Boxplots show controls, positive controls, Inzecto and In2Care results. Individual water samples are shown as dots. Box plots display the median (center line), interquartile range (25th–75th percentile; box), and 1.5× IQR (whiskers), with all individual data points overlaid.

Relative to the control, emergence was significantly reduced in both the In2Care (estimate = 1.892, *z* = 6.50, *P* < 0.0001) and INZECTO (estimate = 0.902, *z* = 3.23, *P* = 0.011) treatments in the case of both species (Fig. 3). This corresponds to substantially lower odds of emergence under both treatments, with a stronger effect for In2Care which reduced emergence significantly more than INZECTO (estimate = −0.990, *z* = −3.40, *P* = 0.0062).

The positive control treatments exhibited near-zero emergence across replicates (Fig. 3). When included in the model, these groups produced extremely large coefficient estimates and standard errors, consistent with quasi-complete separation, and were therefore not included in formal pairwise comparisons. Nevertheless, emergence in positive controls was effectively absent, indicating a complete or near-complete reduction relative to all other treatments.

For *Cx. quinquefasciatus*, both treatments reduced emergence relative to controls. In2Care significantly reduced emergence compared to the control (*P* = 0.0003), and INZECTO also reduced emergence (*P* = 0.0409). In2Care reduced emergence significantly more than INZECTO (*P* = 0.0240).

For *Ae. notoscriptus*, a similar pattern was observed. In2Care significantly reduced emergence relative to both control (*P* < 0.0001) and INZECTO (*P* = 0.0240). INZECTO also reduced emergence relative to control (*P* = 0.0412).

### Survival Data

**Fig. 4.**
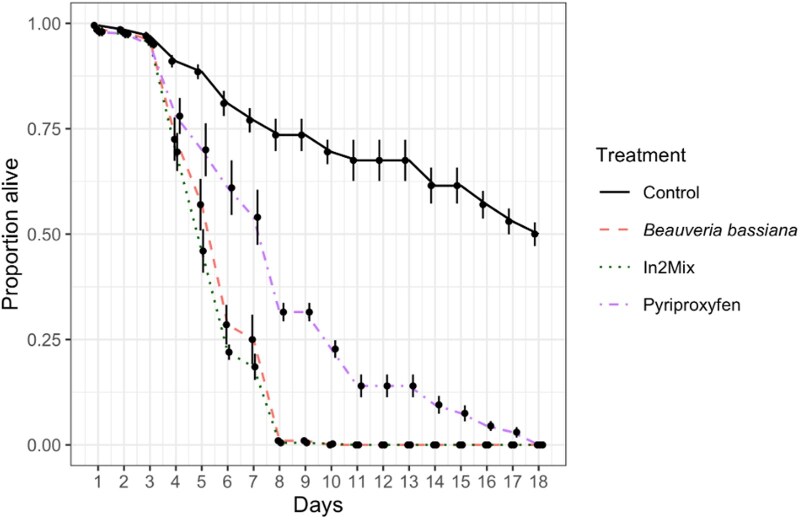
Survival of adult *Aedes notoscriptus* after exposure to nettings carrying different actives. Exposure to *Beauveria bassiana* is shown in red, while exposure to pyriproxyfen is shown in purple, In2Mix is shown in light blue, and the control exposure is shown in green. Data shows the averaged proportion of survival from *n* = 8 replicates per group. Error bars indicate standard errors.

A Cox proportional hazards regression model was used to assess the effect of different treatments on mosquito survival over time (Fig. 4). The model revealed significant differences in survival between the control group and the treatment groups (*B. bassiana*, In2Mix, and Pyriproxyfen; likelihood ratio test = 594.1, *df* = 3, *P* < 2e-16).

Exposure to *B. bassiana*-only was associated with a 7.26-fold increase in mortality hazard relative to the control (HR = 7.26, 95% CI: 5.85–9.02, *P* < 2e-16). Similarly, In2Mix exposure was associated with an 8.64-fold increase in mortality hazard compared to the control (HR = 8.64, 95% CI: 6.98–10.69, *P* < 2e-16). The PPF treatment group also showed a significantly elevated mortality risk (HR = 2.35, 95% CI: 1.92–2.89, *P* < 2e-16), corresponding to more than double the hazard of death relative to the control. The concordance index for the model was 0.689, indicating moderate predictive discrimination.

## Discussion

Urban mosquito control requires innovative, targeted interventions that can function effectively within complex habitats, where conventional strategies such as broad-spectrum insecticide applications may be inadequate due to resistance (e.g., Gratz and Jany 1994, McCarroll et al. 2000), unacceptable to communities without strong engagement and participation (Yulfi et al. 2025), or associated with undesirable off-target environmental impacts (e.g., Abeyasuriya et al. 2017, Jobe et al. 2023). Container-breeding species such as *Ae. notoscriptus* and *Cx. quinquefasciatus* exploit small, dispersed water sources that are difficult to detect and treat systematically through source reduction campaigns. Autodissemination strategies, which leverage mosquito behavior to transfer insect growth regulators to oviposition sites, therefore represent a promising approach for improving control efficiency in urban landscapes. In this study, we evaluated both the semifield autodissemination performance of two control station designs (INZECTO and In2Care) and the adulticidal effects of In2Care-based treatments to assess their combined potential for population suppression.

The semifield experiments demonstrated strong treatment effects, with the In2Care stations significantly reducing adult emergence relative to controls and producing a greater reduction than INZECTO OviTraps containing pyriproxyfen chips (Fig. 3). Although both treatments suppressed adult emergence, In2Care exhibited the strongest inhibition. These findings are consistent with effective horizontal transfer of pyriproxyfen from contaminated adults to oviposition sites, a mechanism demonstrated in previous studies (Devine et al. 2009, Caputo et al. 2012). Field-based evaluations of the In2Care system have similarly shown substantial reductions in emergence and evidence of pyriproxyfen transfer to surrounding breeding sites in *Aedes* mosquitoes (Thammavong et al. 2022, Paris et al. 2023), although species-specific contributions to autodissemination are often difficult to resolve under field conditions. Recent semifield work further demonstrates that *Cx. quinquefasciatus* can effectively transfer pyriproxyfen from In2Care stations to surrounding breeding sites (Buckner et al. 2025).

The difference in autodissemination efficacy between the two station designs is likely driven by differences in pyriproxyfen delivery. In the In2Care system, mosquitoes contact electrostatically charged gauze treated with pyriproxyfen, facilitating adherence of the active ingredient to the insect cuticle and subsequent transfer between sites. In contrast, INZECTO OviTraps rely on pyriproxyfen dissolved in water, with exposure occurring primarily during oviposition when mosquitoes may contact the surface of treated water. This mode of exposure likely limits the quantity of pyriproxyfen carried by individual mosquitoes and reduces transfer to alternative breeding sites.

These results should be interpreted in the context of the enclosed semifield tests. The limited number of oviposition sites in the tents and structural restrictions imposed by the tents would have increased repeated contact with the same containers, particularly if these also acted as mosquito resting sites. This would have enhanced autodissemination efficiency relative to open and more heterogeneous field environments. Under real-world conditions, gravid mosquitoes encounter a wider range of breeding habitats, which may dilute contact with treated stations and reduce acquisition and transfer of pyriproxyfen.

The primary pathway evaluated here is the transfer of pyriproxyfen from treated devices to untreated oviposition containers via adult mosquito contact, resulting in suppression of adult emergence. The efficiency of this pathway is likely to be context-dependent and may vary over time. Extended deployment could increase dissemination through repeated contamination events, but efficacy may also decline if pyriproxyfen residues degrade, are washed off, or are taken up less effectively than in the design used here. In addition, environmental conditions within breeding sites are expected to influence performance. Higher organic content, for example, may reduce pyriproxyfen bioavailability through adsorption to particulates or microbial degradation, potentially lowering effective concentrations when compared with the low-organic conditions used in this study.

Autodissemination efficiency is particularly relevant when considering the two species tested here, given the substantial differences in oviposition behavior between *Culex quinquefasciatus*, which typically deposits egg rafts at a single breeding site (Christophers 1945, Day 2016), and *Ae. notoscriptus*, which exhibits skip-oviposition to distribute eggs across multiple containers during a single gonotrophic cycle (Colton et al. 2003). Skip-oviposition should increase the number of sites contaminated by a single female, while single-site oviposition may concentrate pyriproxyfen within fewer habitats.

Despite these expectations, species had a negligible and nonsignificant effect on emergence in this study. This may reflect either a true similarity in autodissemination efficiency across oviposition strategies or restrictions imposed by the semifield conditions tested here. Further evaluation of dissemination efficiency is required under natural field conditions, where there will be a greater diversity of breeding and resting sites.

The positive control treatments exhibited near-zero adult emergence, confirming that the active ingredients were effective and that the experimental setup functioned as intended (Fig. 3). Notably, no emergence was observed in the In2Care positive controls, indicating complete suppression of development. In contrast, low levels of emergence persisted in the INZECTO positive controls (mean ≈ 1.5 individuals per replicate), suggesting that the pyriproxyfen chip formulation did not achieve complete inhibition within the timeframe of the assay. This difference could be related to the slow-release nature of the chips, which require time to reach fully inhibitory concentrations in the water column.

In addition to suppressing emergence, the In2Care system exerted strong adulticidal effects (Fig. 4). Exposure to *B. bassiana* increased the mortality hazard more than seven-fold relative to controls, while In2Mix increased the mortality hazard more than eight-fold. The near-identical survival curves for *B. bassiana* and In2Mix indicate that the fungus was the dominant driver of adult mortality (Fig. 4). *Beauveria bassiana* infects mosquitoes upon contact and proliferates within the host, producing toxins that disrupt physiological function and ultimately cause death (Lacey et al. 2015). Although mortality is delayed, the observed increase in hazard implies a substantial reduction in adult lifespan, likely limiting the number of gonotrophic cycles and reducing the probability of pathogen transmission. Nevertheless, pyriproxyfen also contributed to adult mortality, approximately doubling mortality hazard relative to controls. While weaker than the fungal effect, this contribution may enhance the robustness of the In2Mix formulation. The effect of pyriproxyfen on mortality may persist even if fungal efficacy declines under field conditions, providing complementary modes of action within the system.

## Conclusion

Taken together, the semifield and survival results indicate that the In2Care system reduced recruitment through inhibition of immature development and shortened adult lifespan through fungal infection and, to a lesser extent, pyriproxyfen exposure. INZECTO OviTraps also reduced emergence relative to controls, although the magnitude of suppression was lower under the conditions tested. These findings demonstrate that both systems can reduce mosquito recruitment, with differences in observed efficacy likely reflecting their respective modes of pyriproxyfen delivery and the experimental context. This is particularly relevant in urban environments, where cryptic breeding sites can sustain rapid population recovery following conventional interventions and where sustained or repeated exposure may be required to achieve consistent suppression.

## Supplementary Material

tjag078_Supplementary_Data

## References

[tjag078-B1] Abeyasuriya KGTN , Nugapola NWNP, Perera MDB, et al 2017. Effect of dengue mosquito control insecticide thermal fogging on non-target insects. Int. J. Trop. Insect Sci. 37:250–258.

[tjag078-B2] Bhatt S , Gething PW, Brady OJ, et al 2013. The global distribution and burden of dengue. Nature 496:504–507. 23563266 10.1038/nature12060 PMC3651993

[tjag078-B3] Brisco KK , Jacobsen CM, Seok S, et al 2023. Field evaluation of In2Care mosquito traps to control *Aedes aegypti* and *Aedes albopictus* (Diptera: Culicidae) in Hawai’i Island. J. Med. Entomol. 60:364–372. 36656078 10.1093/jme/tjad005 PMC9989837

[tjag078-B4] Brooks M , Bolker B, Kristensen K, et al 2017. Package “glmmTMB.” R J. 9:378–400.

[tjag078-B5] Buckner EA , Romero-Weaver AL, Schluep SM, et al 2025. Evaluation of the In2Care Mosquito Station against *Culex quinquefasciatus* mosquitoes (Diptera: Culicidae) under semifield conditions. J. Med. Entomol. 61:1–9. 10.1093/jme/tjae124 PMC11735264 39373161

[tjag078-B6] Buckner EA , Williams KF, Marsicano AL, et al 2017. Evaluating the vector control potential of the In2Care^®^ mosquito trap against *Aedes aegypti* and *Aedes albopictus* under semifield conditions in Manatee County, Florida. J. Am. Mosq. Control Assoc. 33:193–199. 28854105 10.2987/17-6642R.1

[tjag078-B7] Buckner EA , Williams KF, Ramirez S, et al 2021. A field efficacy evaluation of In2Care mosquito traps in comparison with routine integrated vector management at reducing *Aedes aegypti*. J. Am. Mosq. Control Assoc. 37:55–64. 10.2987/21-7038 34817613

[tjag078-B8] Campbell LP , Luther C, Moo-Llanes D, et al 2015. Climate change influences on global distributions of dengue and chikungunya virus vectors. Philos. Trans. R. Soc. B Biol. Sci. 370:20140135. 10.1098/rstb.2014.0135 PMC4342968 25688023

[tjag078-B9] Caputo B , Ienco A, Cianci D, et al 2012. The “auto-dissemination” approach: a novel concept to fight *Aedes albopictus* in urban areas. PLoS Negl. Trop. Dis. 6:e1856. 22953015 10.1371/journal.pntd.0001793 PMC3429402

[tjag078-B10] Christophers SR. 1945. Structure of the *Culex* egg and egg-raft in relation to function (Diptera). Trans. R. Entomol. Soc. Lond. 95:17–34.

[tjag078-B11] Colton YM , Chadee DD, Severson DW. 2003. Natural skip oviposition of the mosquito *Aedes aegypti* indicated by codominant genetic markers. Med. Vet. Entomol. 17:195–204. 12823837 10.1046/j.1365-2915.2003.00424.x

[tjag078-B12] Day JF. 2016. Mosquito oviposition behavior and vector control. Insects 7:65. 27869724 10.3390/insects7040065 PMC5198213

[tjag078-B13] Devine GJ , Perea EZ, Killeen GF, et al 2009. Using adult mosquitoes to transfer insecticides to *Aedes aegypti* larval habitats. Proc. Natl. Acad. Sci. USA. 106:11530–11534. 19561295 10.1073/pnas.0901369106 PMC2702255

[tjag078-B14] Dusfour I , Vontas J, David JP, et al 2019. Management of insecticide resistance in the major *Aedes* vectors of arboviruses: advances and challenges. PLoS Negl. Trop. Dis. 13:e0007615. 31600206 10.1371/journal.pntd.0007615 PMC6786541

[tjag078-B15] Elfekih S , AbdulGhaffar M, Gibreel Y, et al 2025. Effective *Aedes* mosquito reduction through In2Care interventions in an extreme environment. J. Med. Entomol. 62:1243–1252. 40590269 10.1093/jme/tjaf081

[tjag078-B16] Gratz NG , Jany WC. 1994. What role for insecticides in vector control programs? Am. J. Trop. Med. Hyg. 50:11–20. 8024077

[tjag078-B17] Hartig F. 2024. DHARMa: residual diagnostics for hierarchical (multi-level/mixed) regression models. R Package Version 0.4.6. https://CRAN.R-project.org/package=DHARMa.

[tjag078-B18] Itoh T , Kawada H, Abe A, et al 1994. Utilization of bloodfed females of *Aedes aegypti* as a vehicle for the transfer of the insect growth regulator pyriproxyfen to larval habitats. J. Am. Mosq. Control Assoc. 10:344–347. 7807075

[tjag078-B19] Jobe NB , Huijben S, Paaijmans KP. 2023. Non-target effects of chemical malaria vector control on other biological and mechanical infectious disease vectors. PLoS Negl. Trop. Dis. 17:e0011471. 10.1016/S2542-5196(23)00136-5 37558351

[tjag078-B20] Kancharlapalli SJ , Crabtree CJ, Surowiec K, et al 2021. Indirect transfer of pyriproxyfen to European honeybees via an autodissemination approach. PLoS Negl. Trop. Dis. 15:e0009365. 34648501 10.1371/journal.pntd.0009824 PMC8516248

[tjag078-B21] Lacey LA , Grzywacz D, Shapiro-Ilan DI, et al 2015. Insect pathogens as biological control agents: back to the future. J. Invertebr. Pathol. 132:190–206. 26225455 10.1016/j.jip.2015.07.009

[tjag078-B22] McCarroll L , Paton MG, Karunaratne SHPP, et al 2000. Insecticides and mosquito-borne disease. Nature 407:961–962. 11069167 10.1038/35039671

[tjag078-B23] Paris V , Bell N, Schmidt TL, et al 2023. Evaluation of In2Care mosquito stations for suppression of the Australian backyard mosquito, *Aedes notoscriptus* (Diptera: Culicidae). J. Med. Entomol. 60:1061–1072. 37535973 10.1093/jme/tjad099 PMC10496431

[tjag078-B24] R Core Team. 2021. R: a language and environment for statistical computing. R Foundation for Statistical Computing, Vienna, Austria. https://www.R-project.org/.

[tjag078-B25] Ross PA , Axford JK, Richardson KM, et al 2017. Maintaining *Aedes aegypti* mosquitoes infected with *Wolbachia*. J. Vis. Exp. 126:e57930. 10.3791/56124 PMC5614331 28829414

[tjag078-B26] Suleman M , Shirin M. 1981. Laboratory studies on oviposition behaviour of *Culex quinquefasciatus* Say (Diptera: Culicidae): choice of oviposition medium and oviposition cycle. Bull. Entomol. Res. 71:93–101.

[tjag078-B27] Tatem AJ , Gething PW, Smith DL, et al 2013. Urbanization and the global malaria recession. Malar. J. 12:133. 23594701 10.1186/1475-2875-12-133 PMC3639825

[tjag078-B28] Thammavong P , Boyer S, Luangamath P, et al 2022. Small-scale field assessment against the dengue vector *Aedes aegypti* using the autodissemination approach in an urban area of Vientiane, Lao PDR. PLoS One. 17:e0270493. 35776762 10.1371/journal.pone.0270987 PMC9249186

[tjag078-B29] Therneau TM , Lumley T. 2015. Package ‘survival’: a package of survival analysis functions. R Package Version 2.38. https://CRAN.R-project.org/package=survival.

[tjag078-B30] Thieurmel B , Elmarhraoui A, Thieurmel MB. 2019. Package ‘suncalc’. R Package Version 0.5, 1.

[tjag078-B31] Trewin B , Pagendam DE, Darbro JM, et al 2020. Urban landscape features influence the movement and distribution of the Australian container-inhabiting mosquito vectors *Aedes aegypti* (Diptera: Culicidae) and *Aedes notoscriptus*. J. Med. Entomol. 57:443–453. 31693154 10.1093/jme/tjz187

[tjag078-B32] Unlu I , Rochlin I, Suman DS, et al 2020. Large-scale operational pyriproxyfen autodissemination deployment to suppress the immature Asian tiger mosquito (Diptera: Culicidae) populations. J. Med. Entomol. 57:1120–1130. 32006427 10.1093/jme/tjaa011 PMC7448106

[tjag078-B33] World Health Organization. 2006. Guidelines for testing mosquito adulticides for indoor residual spraying and treatment of mosquito nets. World Health Organization. https://www.who.int/docs/default-source/ntds/vector-control/who_cds_ntd_whopes_gcdpp_2006.3.pdf.

[tjag078-B34] Yulfi H , Panggabean M, Darlan DM, et al 2025. Community-based intervention in mosquito control strategy: a systematic review. Narra J. 5:e1234. 10.52225/narra.v5i1.1015 PMC12059817 40352237

